# Identification of a new mutation in the human xanthine dehydrogenase responsible for xanthinuria type I

**DOI:** 10.1515/almed-2021-0018

**Published:** 2021-07-21

**Authors:** Cristina Collazo Abal, Susana Romero Santos, Carmen González Mao, Emilio C. Pazos Lago, Francisco Barros Angueira, Daisy Castiñeiras Ramos

**Affiliations:** Clinical Analysis Department, University Hospital of Vigo, Vigo, Spain; Internal Medicine Department, University Hospital of Vigo, Vigo, Spain; Galician Public Foundation for Genomic Medicine, University Hospital of Santiago de Compostela, Santiago de Compostela, Spain; Laboratory of Metabolic Pathologies, University Hospital of Santiago de Compostela, Santiago de Compostela, Spain

**Keywords:** hypouricemia, xanthine dehydrogenase gene, xanthinuria

## Abstract

**Objectives:**

Hereditary xanthinuria is a rare, autosomal and recessive disorder characterized by severe hypouricemia and increased xanthine excretion, caused by a deficiency of xanthine dehydrogenase/oxidase (XDH/XO, EC: 1.17.1.4/1.17.3.2) in type I, or by a deficiency of XDH/XO and aldehyde oxidase (AOX, EC: 1.2.3.1) in type II.

**Methods:**

We describe a novel point mutation in the *XDH* gene in homozygosis found in a patient with very low serum and urine levels of uric acid, together with xanthinuria. He was asymptomatic but renal calculi were discovered during imaging.

**Results:**

Additional cases were found in his family and dietary recommendations were made in order to prevent further complications.

**Conclusions:**

Hereditary xanthinuria is an underdiagnosed pathology, often found in a routine analysis that shows hypouricemia. It is important for Laboratory Medicine to acknowledge how to guide clinicians in the diagnosis.

## Introduction

Hereditary xanthinuria (HX) is a rare disorder of purine catabolism caused by an inherited xanthine dehydrogenase/oxidase (XDH/XO, EC: 1.17.1.4/1.17.3.2) deficiency, first described in 1954 by Dent and Philpot [1]. It is characterized by very low or undetectable levels of uric acid in blood and urine, with a normal or low fractional excretion of urate together with elevated excretion of xanthine and hypoxanthine in urine. Although most patients are asymptomatic, possible clinical symptoms include urolithiasis due to increased excretion of xanthine and myositis for xanthine deposition. No specific treatment is available, but a low purine diet and hydration must be recommended to the patient to prevent further complications.

**Figure 1:**
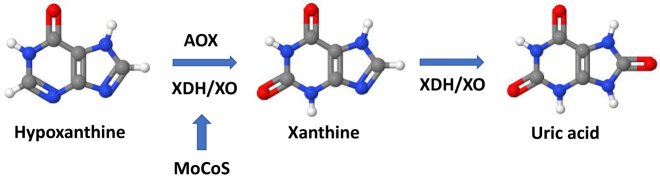
Uric acid metabolism.

Xanthinuria is an autosomal and recessive disorder that consists of three subgroups. Type I (OMIM 278300) is caused by a human XDH/XO deficiency, an enzyme which catalyzes the hypoxanthine oxidation to xanthine, and the formation of uric acid from xanthine, due to mutations in the XDH/XO gene localized on chromosome 2p23 [2], [3], [4], [5], [6] (Figure 1). Additionally, mutations in the molybdenum cofactor sulfurase gene (MOCOS, EC: 2.8), mapped to chromosome 18q12.2, raise xanthinuria type II (OMIM 603592), developing both XDH/XO and aldehyde oxidase (AOX) deficiency. Both types I and II have a similar phenotype, with patients generally asymptomatic, and are referred to as classical HX [7]. Nevertheless, in the xanthinuria type III, there is a very different phenotype, associated with progressive neurological damage due to failure of molybdenum cofactor biosynthesis pathway that causes loss of enzymatic activity for XDH/XO, AOX, and sulfite oxidase [8]. The vast majority of these patients suffer a premature death and the ones that survive present convulsions, abnormal muscle tone, developmental delay, and crystalline lens luxation.

Different mutations described during the last decade associated with xanthinuria type I and II have been reviewed by Ichida et al. [3], and other mutations have been described recently [5, 9, 10]. We report below a new mutation of XDH/XO associated with a case of xanthinuria type I.

## Materials and methods

### Patient

A 65-year-old man was found to have severe hypouricemia in routine analysis, present for at least 4 years. Physical findings were height, 167 cm; body weight, 73.5 kg, body mass index, 26.4 kg/m^2^; blood pressure, 138/90 mmHg; and pulse rate, 90 beats per minute. Physical examination showed no abnormal findings and his medical history included revascularization due to chronic ischemic heart disease and a bilateral inguinal herniography. His parents were not consanguineous and there was no history of urinary calculi in his family. His usual medication was bisoprolol, atorvastatin/ezetimibe, and pantoprazole. He had never taken any antihyperuricemic agent.

On his laboratory data, the serum levels of uric acid were less than the sensitivity of the technique (<5.95 μmol/L), with a normal renal function (creatinine clearance >90 mL/min/1.73 m^2^). The urine analysis confirmed a low excretion of purines, including uric acid (53.6 μmol/24 h).

A renal echography revealed both kidneys with normal size, morphology, and echostructure. In the right kidney, two hypoechoic images (7 and 9 mm) were identified as suggestive of renal lithiasis without repercussion over the excretory system.

**Figure 2:**
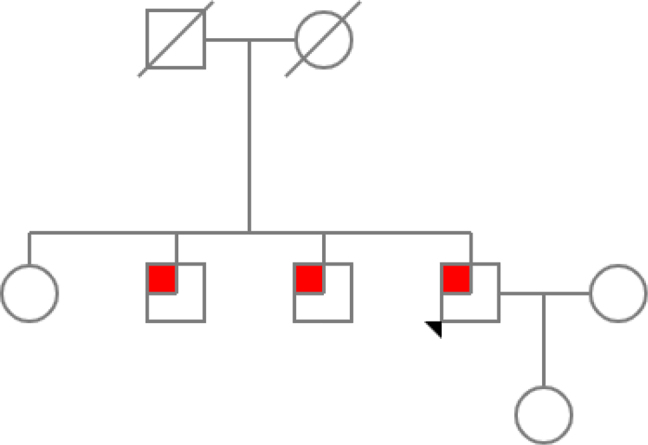
Family tree.

His first-degree relatives were tested, finding that at least two of his siblings had low levels of uric acid (Figure 2). One of them agreed to have his 24 h urine tested, obtaining similar xanthine and uric acid levels (Table 1).

**Table 1:** Purine and pyrimidine results obtained in 24 h urine.

Purines and pyrimidines	Reference interval, µmol/mmol creatinine	Patient	Brother
Uracile	3.26–28.72	1.05	1.39
Thymine	3.29–22.46	0.34	0.41
Hypoxanthine	7.94–92.95	14.44	17.00
Xanthine	0.02–103.35	108.35	117.41
Uric acid	615.34–5,712.26	12.15	15.86
Uridine	29.21–181.00	0.76	0.73
Deoxyadenosine	2.84–33.74	10.89	17.10
Adenosine	2.46–11.30	0.29	0.25
Deoxyguanosine	0.35–5.07	0.05	0.05
Inosine	0.48–15.62	0.59	0.45
Guanosine	0.41–8.16	0.15	0.08
Deoxyuridine	1.45–31.21	0.25	0.52
Thymidine	1.37–21.49	0.17	0.21

### Determination of compounds in serum and urine

Serum and urinary uric acid levels were quantified by a uricase method (Advia 2400; Siemens Medical Solutions Diagnostics, Los Ángeles, CA, USA) and confirmed by a urate oxidase method (Dimension EXL; Siemens Medical Solutions Diagnostics, Los Ángeles, CA, USA). Urinary levels of purines and pyrimidines were measured in a 24 h urine dried sample by tandem mass spectrometry (API 4000 Sciex Applied Biosystems) [11].

Urinary levels of *N*1-methyl-2-pyridone-5-carboxamide (2PY) and *N*1-methyl-4-pyridone-5-carboxamide (4PY) were measured by ultra performance liquid chromatography (UPLC) at the Purine Research Laboratory (St Thomas’ Hospital NHS Foundation Trust, London, UK).

### Genetic analysis – Next-Generation Sequencing (NSG)

The NSG assay was performed using the SureSelectXT Custom kit to capture the coding and intronic flanking regions of the XDH and MOCOS genes. Barcoded samples were pooled, and captured libraries were sequenced on an Ion Proton System using the Ion PI HiQ Sequencing kit and the Ion PI Chip (ThermoFisher Scientific). Sequences were aligned against the human genome sequence (build GRCh37/hg19) with a Torrent Mapping Alignment program. After sequence mapping, the variants were collected and annotated using the following software packages: Torrent Variant Caller (TVC 5.2–25), GATK v3.7-0, Picard 2.9.0-1-SNAPSHOT, BEDtools v2.26.0, SAMtools 1.4, ExomeDepth 1.1.10.

## Results

### Determination of compounds in serum and urine

Serum levels of uric acid were less than 5.95 μmol/L (reference interval: 142.75–356.88 μmol/L). The urinary excretion of uric acid was 12.15 μmol/mmol creatinine (reference interval: 615.34–5,712.26 μmol/mmol creatinine). The daily urinary excretion of xanthine and hypoxanthine were 108.35 μmol/mmol creatinine (reference interval: 0.02–103.35 μmol/mmol creatinine) and 14.44 μmol/mmol creatinine (reference interval: 7.94–92.95 μmol/mmol creatinine), respectively.

Urinary levels of 2PY were of 15.9 μmol/mmol creatinine in a second sample but it was not possible to measure 4PY, possibly because of dilution of the second urinary sample obtained (1.7 mmol creatinine/L).

### Genetic analysis – Next-Generation Sequencing

The patient was found to be homozygous for a point mutation of G to C in the −1 position of the intron 23 of the XDH gene (NM_000379.3(XDH):c.2545-1G>C). This is a new point mutation in the human XDH gene considered as pathogenic using American College of Medical Genetics and Genomics (ACMG) criteria [12]. This mutation present in an intron could affect the splicing process, modifying the RNA transcription, giving, as a result, an XDH deficit and our patient’s phenotype. We did not find any other mutation in this gene nor in the MOCOS gene, which excluded a xanthinuria type II diagnosis.

## Discussion

The annual estimated incidence of HX is from 1:6,000 to 1:69,000, with over 150 patients diagnosed [13]. Just a few cases have been described in Spain where there should be an annual incidence superior to 600 cases with the actual demographic data [14–17]. This only proves that it is an underdiagnosed pathology, often found in a routine analysis that shows hypouricemia.

The diagnosis of HX is made by extremely low serum and urinary uric acid together with high excretion of xanthine, once other causes are discarded. The differential diagnosis of hypouricemia is based on the proportion of uric acid excretion [18]. On the one hand, the possible causes of a patient having hypouricemia and elevated uric acid excretion include treatment with salicylates, intravenous contrast dye, parental nutrition, neoplasia, Wilson’s disease, syndrome of inappropriate antidiuretic hormone, Fanconi’s syndrome, cystinosis, myeloma, heavy metals, hereditary renal hypouricemia, and diabetes mellitus. All these were dismissed in our patient as it had a very low uric acid excretion [19, 20]. On the other hand, low levels of serum uric acid together with a low excretion has been described in neoplasms, severe hepatopathies, treatment with XO inhibitors, and xanthinuria. The patient was asymptomatic with normal hepatic enzymes values, and he referred never had been treated with hypouricemic drugs. This information guided the diagnosis to a new case of xanthinuria.

Secondly, an allopurinol loading test has been traditionally used to determine the type of HX [21]. In healthy volunteers, oxypurinol is excreted in urine after allopurinol administration as it is metabolized by both XDH/XO and AOX. On the contrary, in patients with xanthinuria type II, there is no detection of oxypurinol in urine or in serum as there is no activity for none of them. By contrast, in cases with xanthinuria type I, oxypurinol can be detected as there is still activity for AOX, although there is not for XDH/XO. Finally, confirmation of HX can be supported by measuring XDH/XO activity in the duodenal mucosa. Very low levels of XDH activity in the duodenal mucosa would confirm the diagnosis of xanthinuria. This option was ruled out considering the invasive nature of the procedure.

It has recently been described as a three-step noninvasive diagnostic algorithm [10]. The first step of diagnosis of HX is based on low levels of uric acid determined in serum and urine and a high concentration of xanthine in urine. The second step consists of typing HX using urinary metabolomics. It has been described that AOX participates in the catabolism of *N*1-methylnicotinamide and that the final metabolites 2PY and 4PY could be used as biomarkers to distinguish HX type I from type II. Patients with HX type II have low levels of 2PY and 4PY as there is no AOX activity [22]. The final step is the confirmation of a mutation in the XDH/XO or MOCOS gene.

In our case, the hypouricemia and low excretion of uric acid were verified by two different methods and we demonstrated the excretion of 2PY, which supports the diagnosis of HX type I. Finally, this diagnosis was confirmed by the homozygous mutation described above.

Furthermore, hypouricemia detected in two of his siblings supports the hereditary component of his condition, although they refused to confirm their results by molecular genetics. Taking all together, we could make a premature diagnosis in this patient, preventing renal complications from the renal calculi he already had and providing him, and his relatives, appropriate diet instructions and adequate monitoring.

## Conclusions

We report here a case of xanthinuria type I due to the homozygous mutation NM_000379.3(XDH):c2545-1G>C. This is a new point mutation that is suggested to be responsible for the patient’s phenotype, although additional xanthinuria cases are needed to confirm this association.
